# Leveraging AI in digital one health: an inter-university collaboration for emerging and re-emerging infectious disease control in Cameroon

**DOI:** 10.3389/fdgth.2025.1507391

**Published:** 2025-06-17

**Authors:** Elvis Asangbeng Tanue, Denis L. Nkweteyim, Moise Ondua, Ginyu Innocentia Kwalar, Odette Dzemo Kibu, Madeleine L. Nyamsi, Peter L. Achankeng, Christian Tchapga, Justine Ayuk, Patrick Jolly Ngono Ema, Maurice Marcel Sandeu, Gregory Eddie Halle-Ekane, Jude Dzevela Kong, Dickson Shey Nsagha

**Affiliations:** ^1^ Department of Public Health and Hygiene, Faculty of Health Sciences, University of Buea, Buea, Cameroon; ^2^ DigiCare Cameroon Consortium, University of Buea, South West Region, Cameroon; ^3^ Department of Computer Science, Faculty of Science, University of Buea, Buea, Cameroon; ^4^ Department of Microbiology and Infectious Diseases, School of Veterinary Medicine and Sciences, University of Ngaoundéré, Ngaoundéré, Cameroon; ^5^ Department of Women and Gender Studies, Faculty of Social and Management Sciences, University of Buea, Buea, Cameroon; ^6^ Department of Genetics and Biostatistics, School of Veterinary Medicine and Sciences, University of Ngaoundéré, Ngaoundéré, Cameroon; ^7^ Department of Obstetrics and Gynaecology, Faculty of Health Sciences, University of Buea, Buea, Cameroon; ^8^ Department of Mathematics & Statistics, York University, Toronto, ON, Canada

**Keywords:** artificial intelligence, inter-university collaboration, emerging infectious diseases, re-emerging infectious diseases, digital one health

## Abstract

Emerging and re-emerging infectious diseases (ERID) pose ongoing threats to global public health, demanding advanced detection methods for effective outbreak mitigation. This article explores collaboration between research teams based in the faculties of Health Sciences and Science of the University of Buea and the School of Veterinary Medicine and Science of the University of Ngaoundere (DigiCare Cameroon) for integrating artificial intelligence (AI) for early detection and management of ERID through a Digital One Health (DOH) approach. DigiCare is part of an interdisciplinary network called Artificial Intelligence for Pandemic and Epidemic Preparedness and Response Network (AI4PEP) aimed at addressing pandemic and epidemic preparedness and response by strengthening more equitable and effective public health preparedness and response to infectious disease outbreaks in low- and middle-income countries. DigiCare is aimed at improving the health and well-being of the population through sustainable and effective solutions that protect lives and ensure a resilient future leveraging on the power of AI and DOH. DigiCare Cameroon was launched on November 23rd, 2023, at the University of Buea campus during an event graced by numerous high-ranking university and government officials from the public health, environmental, scientific research, and veterinary sectors, alongside representatives from civil society, researchers, students, and community leaders. Baseline data have been collected in communities to provide an evidence-based platform to develop applications that tailor AI towards health care delivery using integrated DOH approaches. This inter-university collaboration will ultimately contribute in strengthening the capacities of health systems to prepare, prevent and mitigate epidemics and pandemics.

## Introduction

In recent years, emerging and re-emerging infectious diseases (ERIDs) have caused great disease burdens worldwide (1), and continue to pose significant challenges to public health systems globally, and Cameroon is no exemption. Emerging infectious diseases are diseases that have not occurred in humans before, have occurred previously in humans but affected only small populations in isolated areas, or have occurred in the past but were only recently recognized as distinct diseases caused by infectious agents (2). On the other hand, re-emerging infectious diseases are infectious diseases that created significant health problems in a particular geographic area or globally during a previous time period, then declined greatly, but are now again becoming health problems of major importance (2). These diseases are responsible for a significant proportion of the infectious disease outbreaks that have plagued humanity over the ages (3). Most ERIDs have a zoonotic origin, demonstrating that the diseases have emerged from animals and crossed the species barrier to infect humans (4). The majority of these zoonoses originate from wildlife, while others come from domesticated animals and intensive animal farming (5). Zoonotic diseases are transmitted from animals to humans through direct contact, droplets, water, food, vectors, or fomites (6). These diseases continue to affect communities, highlighting the need for integrated approaches that tackle the interconnectedness of human, animal, and environmental health.

A One Health approach is progressively being considered the most effective way of managing the threats posed by ERIDs (7) as it is founded on the recognition that human, animal and environmental health are interdependent (8). Animal species are noted to provide a shared reservoir for pathogen exchange and spread, and many ERIDs are driven by diverse and active human-animal interactions (9). The One Health approach incorporates the biomedical and social sciences devoted to the study of human disease to those devoted to non-human disease and ecological concerns (10). Inter-disciplinary collaborative research is required to tackle the growing threats from ERIDs. Moreover, a holistic One Health framework is central in detecting and responding to health challenges caused by emerging and re-emerging pathogens (11). One Health is popular for the optimization of how people collaborate towards the health of humans, animals and their shared environment but currently fails to focus on how these data streams integrate (12).

**Figure 1:**
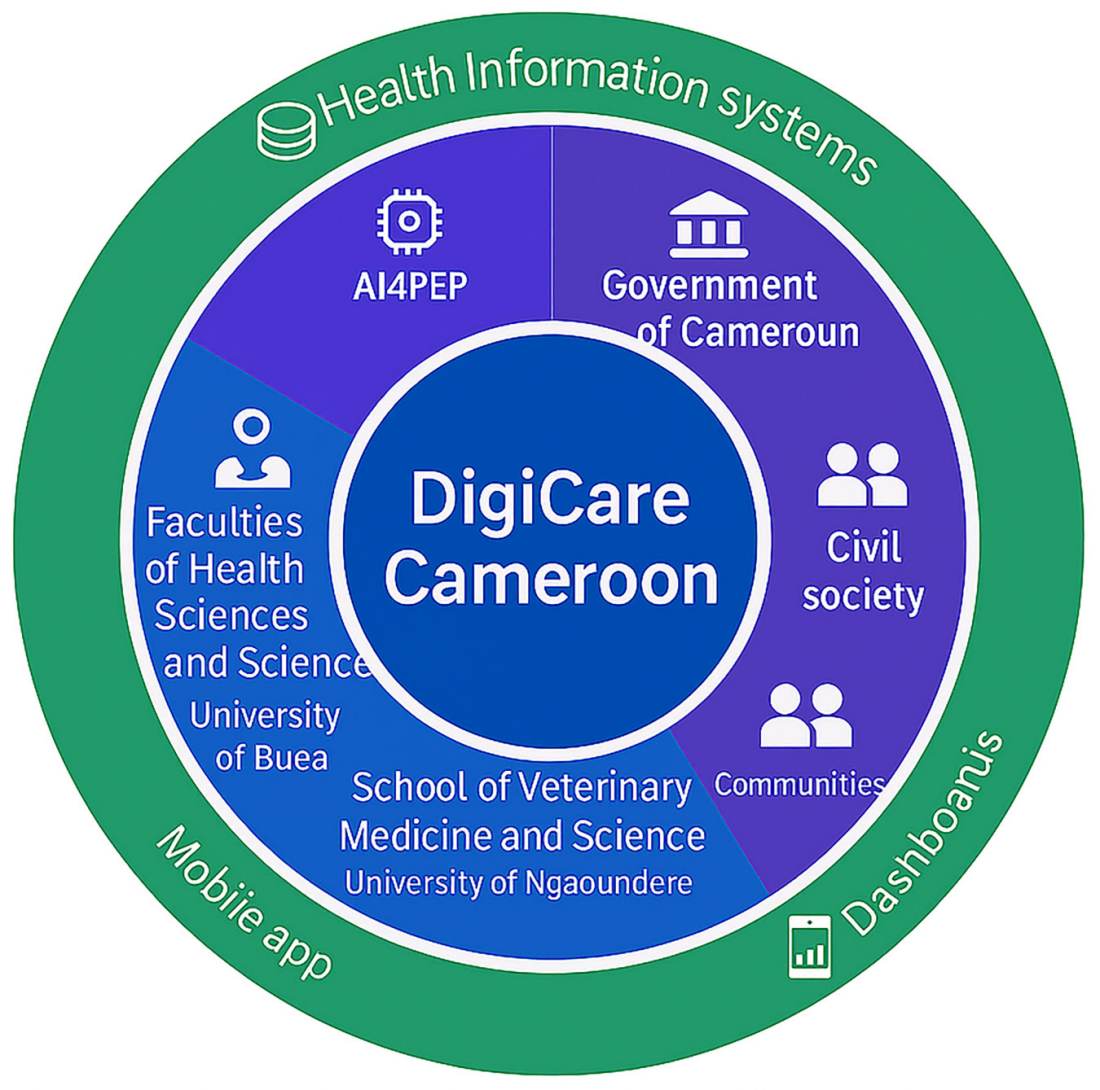
Conceptual framework for DigiCare Cameroon.

In this age of pandemic crisis, Digital One Health is an exciting and optimistic path forward for leveraging technology to create a shared data resource for decision-making for the protection and flourishing of planetary health and wellbeing. In recent years, artificial intelligence (AI) has emerged as a powerful tool in the field of healthcare and has shown great potential in integrating data (13). The integration of AI techniques with conventional disease control strategies offers novel prospects for understanding, predicting, and mitigating the impact of zoonotic diseases (7). By leveraging advanced algorithms and machine learning (ML) models, AI can analyze vast amounts of complex data from diverse sources, ranging from environmental sources to genetic sequences, enabling researchers and public health authorities to make more informed decisions and implement proactive measures (8, 9). This article explores the potential of inter-university collaboration in Cameroon to leverage AI in Digital One Health initiatives aimed at controlling infectious diseases, ranging from zoonotic diseases like Ebola Virus disease to bacterial infections such as cholera. Figure 1 depicts the conceptual framework guiding the collaborations existing between DigiCare, its partners and digital outputs.

## International collaboration

This collaboration is part of an inter-disciplinary network called artificial intelligence for pandemic and epidemic preparedness and response (AI4PEP) aimed at addressing pandemic and epidemic preparedness and response by strengthening more equitable and effective public health preparedness and response to infectious disease outbreaks in low- and middle-income countries. The A4PEP network is a combination of research teams working on two sustainable development goals of SDG3 (“Good Health and Well-being”) and SDG5 (“Gender Equality”). It is built around four research themes: early detection, early warning systems, early response, and mitigation and control of developing epidemics with AI being the entry point. The use AI will improve early detection of outbreaks by analyzing real-time data from sources like social media and patient-level data. Our AI-driven predictive models will combine diverse data sources to forecast outbreaks serving as early warning systems. With the integration of AI, resource allocation and intervention planning such as contact tracing apps will be enhanced thereby fostering early response strategies for health systems. The AI models will be useful in the mitigation and control of misinformation to support public health decision making. These four areas are supported by three pillars: (i) timely and reliable data for public health decision-making, (ii) resilient, strong, and fair health systems and (iii) inclusion and equity for vulnerable groups. These pillars form the backbone of effective AI-driven epidemic management. Timely, accurate data, enabled by interoperable systems and digital tools ensures actionable insights for decision-making. The approach will support the health systems to leverage AI to predict surges and optimize resources. Through inclusion and equity, the teams will use community participatory approaches, to design digital health solutions to ensure vulnerable populations are prioritized, fostering trust and accessibility. Digital One Health is a unified approach that integrates and combines all these domains by leveraging task-specific narrow AI systems to synthesize data from the interconnected domains of human health, animal health, environmental monitoring, and digital ecosystems to enable data-driven strategies for infectious disease prevention, prediction, and response. The partnership started between York University in Canada and Researchers in Africa through the artificial intelligence Global South AI4PEP network. The African researchers are drawn across institutions in 5 countries from Ethiopia (Jimma University), Ghana (Kwame Nkrumah University of Science and Technology), Senegal (Cheikh Anta Diop University), South Africa (University of the Witwatersrand), and Cameroon (University of Buea and University of Ngaoundere) to foster African-led scientific collaboration.

## National collaboration

The project called “artificial intelligence applications to support epidemic and pandemic prevention, preparedness, and response (AI4PEP); controlling re-emerging and emerging infectious diseases using a Digital One Health approach in Cameroon”, tagged “DigiCare Cameroon” is funded by the International Development Research Centre (IDRC) Canada through partnership with York University. The grant (Grant Number: 109981-001) for this project was sub-awarded to the DigiCare Cameroon consortium comprising three research teams from the University of Buea (Faculty of Health Sciences and Faculty of Science) and the Faculty of Veterinary Medicine and Science of the University of Ngaoundere. The governance structure of DigiCare Cameroon comprises a Chairperson, a Vice Chairperson, and a Secretary General; the three principal investigators from the three teams are responsible for managing these positions. Currently, the positions of Chairperson, Vice Chairperson and Secretary General are held respectively by the PIs of the teams at Faculty of Health Sciences, University of Buea, the Faculty of Science and Veterinary Medicine, University of Ngaoundere, and the Faculty of Science, University of Buea.

## Creation of the consortium

The DigiCare Cameroon consortium was created during the selection phase of projects to be funded following a call by AI4PEP. Reviewers in their wisdom recommended that the three projects that were independently submitted by the teams from Cameroon that currently constitute DigiCare could be conveniently placed under a Digital One Health umbrella. The teams concerned accepted this recommendation, and so DigiCare Cameroon was born. Five researchers constitute each team in the consortium as follows: Faculty of Health Sciences at the University of Buea: two Epidemiologist, one Public Health Scientist, one Data Scientist and one Computer Scientist. Faculty of Science at the University of Buea: two Computer Scientists, one Maternal and Child Health Specialist, one Expert on Women and Gender Studies, and one Cardiologist. The team from the Faculty of Veterinary Medicine and Science at the University of Ngaoundere is made up of Veterinarians.

## Diversity of the consortium

DigiCare is diversified in its approach to tackling the problem posed by emerging and re-emerging infectious diseases through the lens of the One Health concept recognizing the interconnection between people, animals and their shared environment. The diversity within the consortium is enriched by the collaboration of various specialists, each contributing unique perspectives and expertise. This collaborative model not only enhances the effectiveness of health interventions but also ensures that the needs of rural and urban populations are met in a comprehensive and equitable manner. By embracing this interdisciplinary approach, DigiCare Cameroon exemplifies the One Health philosophy, ultimately contributing to improved health outcomes across Cameroon and the entire global south. The epidemiologists bring extensive experience to monitor and analyze the distribution of ERID, providing critical insights for outbreak response and prevention strategies. The public health experts add to the strength of the team to implement community health programs tailored to address the specific needs of the local populations, ensuring a focus on pandemic preparedness and response, advocating for policies that promote health equity and access, particularly in underserved communities. Additionally, the data science inclination of the consortium offers analytics skills necessary to analyze data using machine learning algorithms and predictive modeling, which can forecast disease outbreaks and resource needs. The team is empowered to create visual tools that can help stakeholders to understand complex data patterns, facilitating better decision-making. Furthermore, computer scientists are supporting the consortium in developing digital platforms and applications used by DigiCare Cameroon. The gender experts ensure that health interventions consider gender differences and inequalities, advocating for inclusive policies that address the specific needs of women and marginalized groups. Together with the public health experts, the maternal and child health experts engage with communities to promote maternal and child health education, empowering families to make informed health decisions for their communities. The veterinarians play a key role in control of zoonotic diseases that affect both animal and human populations. These experts collaborate with public health experts to implement a One Health approach, emphasizing the interconnectedness of human, animal, and environmental health.

## Launching of DigiCare Cameroon

DigiCare Cameroon was launched on the 23rd of November 2023 in the campus of the University of Buea, marking a pivotal step toward strengthening digital health capacity in Cameroon. The event, chaired by the representative of the University's Vice-Chancellor brought together university leadership, government officials, health and environmental authorities, and community representatives, underscoring broad-based institutional and regional support.

Key strategic outcomes of the launch event included formalized partnerships between the University of Buea and the University of Ngaoundere, aimed at fostering collaborative research initiatives in Digital One Health. The event equally recorded pledges from regional health and environmental authorities to support data-driven Digital One Health initiatives into community health strategies. The high level of institutional and student engagement demonstrated by the full attendance at Amphitheatre 750 further highlighted the growing momentum around Digital One Health innovation among the academic community. Moving forward, DigiCare Cameroon aims to consolidate these early gains through targeted partnership with the community, expanded research networks, and closer engagement with stakeholders in public health, the environment and veterinary medicine to drive sustainable Digital One Health transformation for the control of emerging and re-emerging infectious diseases.

## Impressions of participants of the project launch

Emerging from the launching were opinions that DigiCare is a timely project that would set the pace for the use of technology to prevent and prepare for emerging and re-emerging infectious diseases in Cameroon and the world at large.

## The way forward after the launching

The teams are engaged in baseline data collection from the community that will provide an evidence-based platform to develop applications to tailor artificial intelligence towards the health care industry in the fight against epidemics and pandemics in the context of One Health. Baseline data is geared towards capturing the readiness and preparedness of the local population to adopt AI tools for pandemic preparedness and response.

## Mission of DigiCare Cameroon

The DigiCare project seeks to improve disease control and prevention in Cameroon through the power of digital innovation and Digital One Health approach. The project strives to harness novel technology such as AI-powered dashboards and mobile apps, promote collaboration among diverse stakeholders, and empower communities through training and community-driven disease surveillance to proactively address emerging and re-emerging infectious diseases. The goal of DigiCare is to improve the health and well-being of the population by providing sustainable and effective solutions that protect lives and ensure a resilient future leveraging on the power of artificial intelligence.

## Application of AI within DigiCare

The project will establish health information systems across three pilot health centres to automate the collection of accurate patient-level data. With appropriate authorization, these data will serve as the foundation for machine learning studies focused on disease prediction, as well as data mining initiatives to uncover patterns in patient information. Additionally, a variety of mHealth tools—primarily chatbots utilizing natural language processing—will be developed across different domains to deliver customized, locally-relevant information to users. Complementing these efforts, personalized mobile applications will also be created to provide health education tools that recommend web pages and resources based on individual user profiles as they navigate through digital information spaces. These mobile applications will further enable patients with infectious diseases to request home health care services from health workers, thereby improving access to care while minimizing public exposure risks. Finally, the project will develop interactive dashboards aimed at visualizing and publicly sharing the health-related statistics that the government of Cameroon collects regularly, enhancing transparency and data-driven decision-making in public health.

## Challenges and mitigation strategies

Implementing AI-driven Digital One Health initiatives in Cameroon may face certain challenges centered on data ethics, governance, and risk management. Issues like informed consent, data privacy, data ownership across institutions, algorithmic bias, community mistrust, and cybersecurity threats could hamper integration efforts if not carefully managed. To overcome these barriers, the consortium will develop a standardized ethical framework, adopt unified data governance protocols based on the principles Findable, Accessible, Interoperable and Reusable (FAIR) to protect privacy. Bias mitigation will involve sourcing diverse datasets and involving local community expertise. Community trust will be built through engagement campaigns, and cybersecurity infrastructure will be strengthened. Active policy engagement with community leaders and national stakeholders will further ensure regulatory alignment.

## Potentials of the inter-university collaboration

DigiCare Cameroon offers the potential to strengthen national research capacity, drive AI innovation, and build a sustainable Digital One Health workforce. By integrating the expertise and resources of the two universities, the consortium will develop localized AI tools for early disease detection, real-time surveillance, and outbreak prediction, improving public health responses across human, animal, and environmental sectors. Over time, the consortium's unified voice can influence national health policies, ensuring that digital health innovations are sustainably embedded into Cameroon's public health infrastructure. The project's impact will be measured across reduced disease burden, AI system performance, community engagement levels, compliance with governance standards, professional capacity building, and influence on national digital health policy.

## Conclusion

The project will contribute to the attainment of Sustainable Development Goal Number 3 and 5 in building the capacities of communities and stakeholders in the preparedness, prevention and mitigation of epidemics and pandemics. This inter-university collaboration offers a unique opportunity to harness AI within the Digital One Health framework to combat emerging and re-emerging infectious diseases. By promoting collaborative research, sharing data, and building capacity, universities can significantly contribute to strengthening healthcare systems and improving outcomes in the country. The consortium offers a unique opportunity to address immediate healthcare needs and also lays the groundwork for a more resilient health system in the global south.

## Data Availability

The original contributions presented in the study are included in the article/Supplementary Material, further inquiries can be directed to the corresponding authors.
